# The relationship of religiosity, spirituality and health in Spanish cancer patients: testing underlying psychological, social and behavioral pathways

**DOI:** 10.1186/s40359-025-03097-x

**Published:** 2025-07-09

**Authors:** David Almaraz, Klaus Baumann, Florentino Moreno Martín, Iván Sánchez-Iglesias, Jesús Saiz

**Affiliations:** 1 Department of Social, Work and Differential Psychology, Complutense University of Madrid, Madrid, Spain; 2 Caritas Science and Christian Social Work, Faculty of Theology, Albert-Ludwig University of Freiburg, Platz Der Universität 3, 79098 Freiburg, Germany; 3 Department of Psychobiology & Behavioral Sciences Methods, Complutense University of Madrid, Madrid, Spain

**Keywords:** Religion, Spirituality, Health, Cancer, Patients, Holistic health, Religion and psychology

## Abstract

**Background:**

Religiosity and spirituality (R/S) are associated with health outcomes in cancer patients, yet the psychosocial and behavioral pathways involved remain insufficiently understood, particularly in Spain. This study examines specific R/S elements and pathways that may contribute to perceived physical health in this population.

**Methods:**

A cross-sectional study was conducted with Spanish cancer patients (*N* = 351), recruited between October 2022 and April 2023. Participants were adults (18–80 years) with a non-terminal cancer diagnosis. The responses were obtained through a questionnaire that included sociodemographic and health variables, as well as validated scales measuring spirituality, religiosity, R/S struggles, psychological factors (gratitude, compassion, negative emotions), social support, health behaviors, and perceived physical health. Path analyses were performed with model fit evaluated using multiple indices (χ^2^, CFI, TLI, RMSEA, SRMR).

**Results:**

The global model did not present a good fit to the data, as did the partial model of religiosity. In contrast, the partial models of spirituality and R/S struggles presented good fit indices and a high explained variance, showing that in both models negative emotions and gratitude act as significant mediating pathways between R/S and perceived physical health.

**Conclusions:**

Despite the limitations caused by the low sample size and the apparent misspecification of the religiosity segment, findings from the partial models suggest that decreased negative emotions and increased gratitude promoted by higher levels of spirituality and lower levels of R/S struggles lead to better perceived physical health in cancer patients. These results highlight the need to integrate spirituality into patient care, often overlooked in Spanish healthcare. Recognizing these pathways could contribute to more holistic care approaches that incorporate R/S dimensions, potentially enhancing oncology patients’ quality of life.

## Introduction

Cancer is one of the world's leading causes of morbidity and mortality, with incidence and mortality rates expected to rise significantly in the coming decades [1]. In Spain, cancer is a primary cause of death, particularly among men [2].

Beyond affecting physical, emotional and social aspects, cancer also encompasses spiritual issues, often stemming from the uncertainties and religious and existential questions that arise in the course of illness [3]. In this context, religiosity and spirituality (R/S) have been identified as key factors in the different stages of cancer [4, 5], prompting efforts to integrate R/S into healthcare to provide patients with the best comprehensive care [6].

### Religion and spirituality in cancer patients

Religion is generally defined as an organized system of beliefs, practices and rituals that facilitate closeness to the sacred or transcendent [7]. In this study, spirituality refers to the search for and relationship with the sacred or transcendent, reflecting Spain’s religious roots [7], though its definition varies, and some perspectives separate it from religion [8].

In any case, different aspects of R/S have been associated with better mental and physical health outcomes in cancer patients [9, 10], including in Spain [11–13]. However, despite this evidence, the pathways through which R/S affects physical health of cancer patients remain unclear [14].

### R/S and physical health: the need for an explanation and better understanding

There is a growing consensus on the need to understand the complex relationships between R/S and health to improve the quality of patient care [15]. Although different approaches coincide in the nature of the variables involved [16], the divergencies between the various models highlight the need to homogenize the different explanations by framing them in a broad theoretical proposal, considering the different aspects of R/S and, therefore, allowing the identification of the health outcomes corresponding to each one and the mechanisms involved [17].

### Harmonizing theoretical diversity: building an integrative model for cancer patients

#### Some basic premises

The first idea guiding this proposal has to do with the nature of the pathways involved in the relationship between R/S and health, which, following Oman and Thorensen [16] are psychological, social and behavioral.

The second basic assumption concerns the conceptualization of R/S. Following various authors, religiosity and spirituality will be treated as related but separate constructs [18, 19]. In this context, religiosity refers to the more institutional, normative, and behavioral aspects of the religious experience, including elements such as private and public religious practices and commitment to an organized system of beliefs. In contrast, spirituality refers to a more internal, subjective, and emotional dimension, centered on the personal experience of transcendence and the relationship or connection with the divine. This distinction allows for the independent assessment of both constructs -religiosity through indicators of religious practices and beliefs, and spirituality through measures of closeness, attachment, or relationship with God- thereby enabling the analysis of their specific effects while acknowledging their interrelationship [18, 20]. Thus, religiosity, being more rooted in social and behavioral aspects, is considered to be more strongly associated with variables of that nature, whereas spirituality, involving a deeper affective dimension, would be more directly linked to emotional variables [21].

The third premise highlights the importance of taking into account the particularities of R/S in cancer patients [17]. A cancer diagnosis can profoundly affect religious and spiritual beliefs, sometimes leading to crises that are associated with poorer health outcomes [22]. Aldwin et al. [18] emphasizes the need to address the negative dimension of R/S, often referred to as R/S Struggles or negative religious coping [23, 24]. Given its equivalence in the literature [23, 24], the term R/S Struggles will be used to capture this dimension referring to feelings of religious despair and abandonment or punishment by God, and its specific effects on health.

### Main variables and their relationship with R/S

There is some degree of agreement that emotions, social support, and healthy behaviors are the main pathways linking R/S to health [7, 18, 25–27]. First, in line with our premises, the self-regulation perspective of Aldwin et al. [18] represents the starting point of the present proposal, by suggesting that most of these pathways are aspects of emotional and behavioral self-regulation.

#### Behavioral and emotional pathways

Healthy behaviors are a key pathway linking R/S and health, involving activities aimed at preventing disease and improving well-being [28]. Major religions have historically promoted healthy lifestyles, influencing behaviors such as diet, physical activity, and substance use [29, 30]. From a self-regulatory perspective, religiosity fosters behavioral self-regulation, encouraging engagement in healthy behaviors that, in turn, influence health.

As an emotional pathway, negative emotions play a crucial role. Patients with physical illness often experience distress, which leads to efforts to regulate negative affect [31], which is strongly associated with improved health outcomes [32, 33]. Spirituality, specifically a close relationship with God, has been associated with improved emotional well-being [13, 34, 35]. Thus, spirituality facilitates emotional self-regulation, relating to better health through decreased negative emotions.

Finally, feelings of abandonment by God have been associated with unhealthy behaviors, such as poor diet and substance use [36, 37], as well as increases in negative emotions [38, 39]. Following Aldwin et al. [18], R/S Struggles would negatively affect health by decreasing healthy behaviors and increasing negative emotionality.

#### Social pathways

Social support has been recognized as a social determinant of health, with numerous studies linking higher levels of support with better physical health [40, 41]. For cancer patients, social support plays an important role in coping and improving quality of life, as longitudinal studies have shown it to be associated with better perceived physical health [42, 43].

Regarding R/S, Nelson [26] differentiates between direct social support (from community) and spiritual social support (perceived support from God). Religious involvement has been linked to greater social support, especially in contexts of illness [44, 45]. In line with Aldwin et al. [18], perceived social support serves as a pathway between religiosity and health.

### The role of positive psychological variables: gratitude and compassion

To deepen the relationship studied, this research incorporates variables identified in previous models, which have been referred to as positive psychological traits [7]. The Positive Religious and Spiritual Development (PRSD) theory, grounded in Positive Psychology, examines how individuals develop habits or behavioral and psychosocial patterns that characterize their search for and connection with the sacred. Although PRSD theory provides a more detailed account of neurobiological and motivational processes, its propositions regarding the dimensions of R/S are compatible with the differentiation proposed here between religiosity and spirituality as overlapping but distinct constructs. Thus, as Davis et al. [46] pointed out, this theory is compatible with other theories that assume R/S is related to health through various psychological, social, and behavioral mechanisms [7, 18], helping to integrate elements of positive psychology into the model. In particular, gratitude and compassion are constructs emphasized in PRSD theory [46] and other models [7, 15, 18] as key positive psychological traits that may mediate the relationship between R/S and health. Some research has indicated that interventions based on these traits could be of particular clinical relevance by improving physical health and overall well-being [47, 48]. Moreover, as discussed below, empirical evidence highlights the importance of these variables in the relationship described.

#### The role of gratitude

As a positive psychological trait, gratitude has been associated with better health outcomes, particularly in individuals facing serious illnesses such as cancer [49, 50]. The belief that everything one has in life is a gift ultimately from God may shape how people relate to Him [51]. Therefore, as some literature shows, gratitude shows stronger associations with variables related to a relationship with God (spirituality) than with those related to religious practices and commitment (religiosity) [52–54]. Furthermore, since R/S Struggles involve feelings of abandonment and anger toward God, these have been linked to lower levels of gratitude [55]. In sum, spirituality and R/S Struggles could influence physical health through their effects on gratitude.

#### The role of compassion

Given its relevance in religious teachings, compassion is closely related to R/S. Specifically, King et al. [56] argue that the relationship with God fosters complex social emotions such as compassion, which is supported by studies showing stronger associations of compassion with spirituality than with religiosity [57, 58]. On the other hand, longitudinal studies have found that compassion toward others predicts improvements in physical well-being [59]. Taken together, this suggests that compassion may be a pathway through which spirituality is related to physical health.

### Hypothesized model

**Fig. 1:**
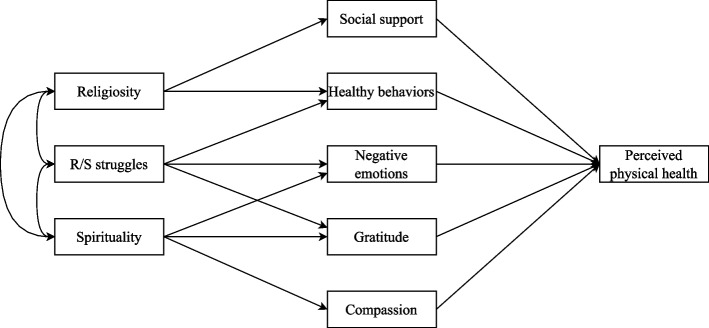
Graphical representation of the hypothesized model

Based on these premises and justifications, this study proposes an integrative model (see Fig. 1) that, considering the interrelationship between religiosity, spirituality, and R/S Struggles, outlines different pathways through which R/S is linked to the patients' perceived physical health: 1) Religiosity influence perceived physical health through social support and healthy behaviors; 2) Spirituality has its impact on perceived physical health through negative emotions, gratitude and compassion; 3) R/S struggles affect perceived physical health through healthy behaviors, negative emotions and gratitude.

### The present study

Conducting this study in Spain is particularly relevant due to the high incidence of cancer, the country's deep Catholic religious tradition alongside growing secularization, and the empirically reported spiritual needs among Spanish cancer patients [60]. The research aims to examine how R/S influences perceived physical health through psychological (negative emotions, gratitude, compassion), social (social support), and behavioral (healthy behaviors) pathways. The hypothesized model is expected to fit the data.

## Method

### Design and setting

This study employed an observational cross-sectional design, analyzing data at a single point in time without longitudinal follow-up. Data were collected between October 2022 and April 2023 through various cancer patient organizations across Spain.

### Participants

The participants were cancer patients who were not in a terminal stage of the disease. A minimum sample size of 344 participants was determined, taking simple random sampling as a reference, and considering the number of people diagnosed with cancer in Spain, a confidence level of 95% and an error of ± 5% were adopted. In the end, the sample consisted of 351 participants, all of whom identified their gender as either woman (n = 209) or man (n = 142), within an age range of 18 to 80 years (M = 52.46; SD = 13.01). Of these, 47.6% had higher education, 43% were currently working and 63.2% belonged to a middle socio-economic level. The most common diagnosis in the sample was breast cancer (35%), with the majority being in a treatment phase (84%) (see Table 1 for more details).

**Table 1:** Socio-demographic characteristics. *N* = 351

Variables		N	%
Age ^a^		52.46	13.01
Gender	Woman	209	59.5
	Man	142	40.5
Education level	No studies	1	0.3
	Elementary education	7	2
	Secondary education	28	8
	Vocational training or high school	96	27.4
	Higher education	167	47.6
	Postgraduate, master’s or doctoral degree	52	14.8
Socioeconomic level	Low	4	1.1
	Lower-middle	52	14.8
	Middle	222	63.2
	Upper-middle	69	19.7
	High	4	1.1
Employment status	Currently working	151	43
	On the dole	20	5.7
	Not working	79	22.5
	Student	19	5.4
	Pensioner/Retired	82	23.4
Main types of cancer	Breast cancer	123	35
	Colon cancer	29	8.3
	Lung cancer	25	7.1
	Lymphoma ^b^	22	6.3
	Prostate cancer	21	6
	Leukemia ^c^	20	5.7
	Ovarian cancer	11	3.1
	Metastasic breast cancer ^d^	11	3.1
	Uterine cancer	9	2.6
	Stomach cancer	9	2.6
Stage of the disease	Treatment	295	84
	In remission	21	6
	Under surveillance	14	4
	Maintenance	2	0.6
	Post-surgery recovery	4	1.1
	Under revision	15	4.3

^a^Mean and Standard Deviation (SD)

^b^Both Hodgkin's and non-Hodgkin's lymphomas

^c^Both myeloid and lymphocytic leukemia

^d^Not part of localized breast cancer above

Individuals aged 18 years or older, of either gender, with a diagnosis of non-end-stage cancer were considered eligible for inclusion in the study. All participants were informed of the characteristics of the study and agreed to participate through informed consent. The study was approved by the Research Ethics Committee of the Complutense University of Madrid (CE_20220616-10_SOC).

### Measures

#### Socio-demographic and health data

An ad hoc survey was used to collect data on age, gender, socio-economic status, educational level, employment status, type of cancer and stage of cancer.

#### Spirituality

The Brief Trust/Mistrust in God Scale by Rosmarin et al. [61] assesses both variables through two subscales of three items each. This instrument has proven to be suitable for the assessment of relationship with God (referred to as spirituality in this study) [7]. It is a Likert-type scale with five response options, from 1 (*not at all*) to 5 (*very much*) (e.g., "No matter how bad things may seem, God’s kindness to me never ceases" or "God loves me immensely"). Each subscale has a minimum possible score of 3 and a maximum of 15. Since the scores of the Mistrust in God subscale were inverted before being summed with those of the Trust in God subscale, the total score ranges from 6 to 30, with higher scores indicating greater spirituality. The subscales of trust in God (α = 0.95) and mistrust in God (α = 0.86) of the Spanish version developed by Almaraz et al. [62] showed good internal consistency.

#### Religiosity

For the assessment of religiosity, we used the DUREL Scale developed by Koenig and Büssing [63]. The first subscale was used to assess public practice (participation in public religious activities and formal religious institutions). It consists of 1 item and is presented on a six-point Likert-type scale from 1 (*not at all*) to 6 (*more than once a week*) (“How often do you attend church or other religious meetings?”). The second subscale measures private practice (frequency of private religious activity). It also comprises 1 item and is assessed on a reverse-coded Likert scale, from 1 (*more than once a day*) to 6 (*never*) (“How often do you spend time in private religious activities, such as prayer, meditation or Bible study?”). Finally, the last subscale, which includes 3 items, assesses religious commitment (importance of people's R/S beliefs in their lives) (e.g., “My religious beliefs are what really lie behind my whole approach to life.”). Each item is rated on a five-point scale from 1 (*definitely not true*) to 5 (*definitely true for me*). Given the mentioned score ranges for each subscale, total scores on the DUREL Scale can vary from 5 to 27, with higher scores indicating greater religiosity. The Spanish adaptation of the DUREL Scale by Taylor [64] showed adequate internal consistency (ranging from α = 0.77 to α = 0.82).

#### R/S struggles

The negative religious coping subscale of the Brief RCOPE by Pargament et al. [24] was used for the assessment of R/S Struggles, given that its items assess feelings of detachment and abandonment by God and the church [23]. It consists of 7 items and is presented in a Likert-type response format with five response options, from 1 (*never*) to 5 (*always*) (e.g., “Felt punished by God for my lack of devotion”). The total score can vary between 7 and 35, with higher values indicating a greater level of R/S Struggles. The reliability of the Spanish version by Rivera-Ledesma and Montero-López [65] was satisfactory, although it was more so for positive religious coping (α = 0.83) than for negative religious coping (α = 0.65).

#### Gratitude

The GQ-6 gratitude questionnaire, developed by McCullough et al. [52], is a self-report scale composed of six items for the measurement of gratitude disposition. It is presented on a seven-point Likert-type scale, with responses ranging from 1 (*strongly disagree*) to 7 (*strongly agree*). Items 3 and 6 have inverse scores. The total score can range from 6 to 42, with higher scores indicating a greater tendency to experience gratitude in everyday life. The Spanish adaptation used, carried out by Beléndez [66], demonstrated adequate internal consistency (ranging from α = 0.76 to α = 0.84).

#### Compassion

The Compassion of Others’ Lives Scale by Chang et al. [67] consists of 26 items, divided between two dimensions. It is a Likert-type response scale from 1 (*does not describe me well*) to 5 (*describes me very well*). In the present study, only 6 items belonging to the alleviating suffering dimension of the Spanish adaptation, developed by Klos and Lemos [68], were used. The internal consistency of this version was very adequate (α = 0.93). The total scores range from 6 to 30, with greater scores reflecting a higher level of compassion towards others.

#### Negative emotions

The negative affect subscale of the Positive and Negative Affect Schedule (PANAS), designed by Watson et al. [69], was used to assess negative emotions. It is a 5-point Likert scale from 1 (*not at all or very slightly*) to 5 (*very much*). A higher score, which ranges from 10 to 50 due to the 10 items in the subscale, indicates a greater presence of negative emotions. The Spanish version of the PANAS, validated by López-Gómez et al. [70] was used, offering adequate reliability (α = 0.88 for negative emotions).

#### Social support

The Multidimensional Scale of Perceived Social Support, created by Zimet et al. [71], is a 12-item scale that assesses perceived level of social support, with three different dimensions. In the present study only items from the “significant others” dimension were used, as it represents an even more abbreviated version to assess perceived social support without focusing on any specific source of support. The scale is presented in Likert format with seven response alternatives, from 1 (*strongly disagree*) to 7 (*strongly agree*). The total score ranges from 4 to 28, with higher scores denotating a greater perceived level of social support. In this study we used the Spanish adaptation made by Ruiz et al. [72], which showed a high reliability (α = 0.94).

#### Healthy behaviors

The Healthy Lifestyles Questionnaire [73] is a valid and reliable instrument to assess the health habits and behaviors of the Spanish population developed by Leyton-Román et al. It is composed of 12 items, which are divided into different factors related to a healthy lifestyle: smoking, sleeping habits, respecting mealtimes and balanced diet. Responses are presented on a five-point Likert-type scale, ranging from 1 (*strongly disagree*) to 5 (*strongly agree*). Since there were missing values in the smoking dimension, the factor structure of the scale was reevaluated, showing adequate structural validity (good data were observed in both the KMO test (0.793) as well as in Bartlett's test of sphericity, χ2(36) = 1404.163, *p* < 0.001). The items were grouped into three components which explained 72.5% of the variance. The total score spans from 9 to 45, with higher scores signifying greater engagement in healthy behaviors. Each factor has adequate internal consistency (ranging from α = 0.71 to α = 0.85).

#### Perceived physical health (Physical and Functional Well-Being)

The FACT-G (Functional Assessment of Cancer Therapy—General), originally developed by Cella et al. [74], is a 27-item instrument that assesses four domains of well-being of cancer patients. The physical and functional well-being subscales were used in this study, as they represent a reliable measure to assess perceived physical health [14]. The instrument uses a five-point Likert scale for recording responses, from 0 (*not at all*) to 4 (*very much*). Both subscales have 7 items each, so the range of scores for each subscale is 0–28. Since the scores from both subscales are summed, the total score range is from 0 to 56, with higher scores indicating a better perceived level of physical health. The items of the physical well-being scale are presented in reverse, so it was necessary to recode them. The reliability of the Spanish version used, adapted by Cella et al. [75] was adequate (α = 0.92).

### Procedure

Various cancer patient organizations throughout Spain were contacted, and a total of 21 organizations agreed to collaborate by providing their patients who met the inclusion criteria with the study information sheet and informed consent. Patients who accepted the informed consent had access to the questionnaire, in physical or online format. The participants then completed the questionnaire anonymously and the responses were collected guaranteeing the rights of the respondents for subsequent data analysis.

Considering the health condition of the participants, several steps were taken to minimize respondent burden. The questionnaire was designed to be completed in a single session lasting approximately 15 to 20 min. Participants were informed that they could take breaks if needed and complete the questionnaire at their own pace. The option to choose between paper and online formats also facilitated accessibility. Although the number of instruments was considerable, no significant difficulties or complaints were reported during the completion process.

### Data analysis

First, descriptive statistics were calculated. Bivariate correlation analyses were performed to evaluate the relationships between variables. Subsequently, a path analysis was performed to test the effects of social support, healthy behaviors, negative emotions, gratitude and compassion on the relationship between R/S variables and perceived physical health. Path analysis is an extension of multiple regression analysis that allows: 1) estimating the relationship between multiple independent and dependent variables, which in turn can operate as independent variables of other variables included in the model; 2) evaluating the fit of the model, generating fit indices that facilitate the comparison of two or more models hypothesized by the researcher [76, 77].

In addition, we compared four path models. A global model was tested using all the variables proposed in Fig. 1. Furthermore, three partial models were specified to explain perceived physical health: a) A model with religiosity as the independent variable, including social support and healthy behaviors as mediators; b) A model with R/S struggles as the independent variable, including healthy behaviors, negative emotions, and gratitude as mediators; and c) a model with spirituality as the independent variable, including negative emotions, gratitude, and compassion as mediators. In each partial model, the residual errors of the mediators were allowed to correlate. This decision is based on the theoretical assumption that unmeasured common factors may lead to correlations among the mediators beyond the shared predictor. Several indices were used to evaluate model fit: chi-square (χ^2^), CFI, TLI, RMSEA, and SRMR. Following Schreiber et al. [78], χ^2^/*df* less than 3, CFI and TLI greater than 0.95, RMSEA 0.06 to 0.08 (with confidence interval), and SRMR equal to or less than 0.08 were considered good fit indices. The level of significance was set at *p* < 0.05. Data were analyzed using SPSS 28 and R.

## Results

### Correlation analysis

All evaluated variables showed significant correlations at the 0.05 level (Table 2).

**Table 2:** Descriptive statistics and correlations between the main variables of the study

Variables	*M*	*SD*	1	2	3	4	5	6	7	8	9
1. Religiosity	12.62	7.75	-								
2. Spirituality	18.36	7.90	.89	-							
3. R/S struggles	13.36	5.46	-.51	-.62	-						
4. Social support	25.85	3.42	.24	.24	-.28	-					
5. HB	38.06	5.69	.36	.35	-.37	.29	-				
6. NE	19.88	6.55	-.51	-.54	.58	-.30	-.53	-			
7. Gratitude	32.63	6.48	.65	.74	-.61	.37	.47	-.68	-		
8. Compassion	23.65	5.00	.42	.50	-.46	.45	.41	-.52	.67	-	
9. PPH	34.15	11.18	.52	.62	-.60	.32	.48	-.73	.72	.53	-

Healthy behaviors, *NE* Negative emotions, *PPH* Perceived physical health. *p* < 0.05

### Path analysis models

#### Global model

**Fig. 2:**
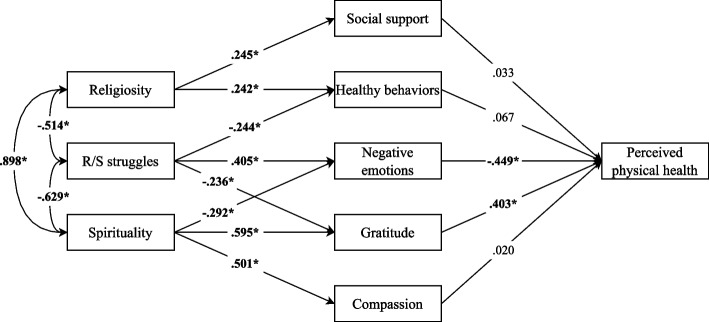
Model results showing the observed pathways between the study variables. Note. The standardized effects between variables are shown. *p < 0.05

The results of the path analysis of the global model showed that religiosity, spirituality and R/S struggles are interrelated (see Fig. 2**)**. On the one hand, positive and significant direct effects of religiosity on social support and healthy behaviors were observed. On the other hand, negative direct effects of spirituality on negative emotions were observed, as well as positive ones on gratitude and compassion. Likewise, R/S struggles had significant negative direct effects on healthy behaviors and gratitude, while these were positive on negative emotions. Finally, only negative emotions and gratitude had significant direct effects on perceived physical health, negative and positive respectively. Although the bivariate correlation of perceived physical health with the other pathways was significant (Table 2), this association disappeared when considering the full model. The model tested (Fig. 2), explained 59% of the variance in perceived physical health (R^2^ = 0.590). However, the model did not present a good fit to the data: χ^2^ = 364.188, df = 20 (*p* < 0.001); CFI = 0.838; TLI = 0.708; RMSEA = 0.221, 90% CI [0.206, 0.247]; SRMR = 0.136.

This poor model fit may be attributed to the complexity of the model (as fit indices tend to penalize models with a high number of estimated parameters), misspecification (namely, the omission of unobserved variables that could better account for the relationships among the included constructs), or a combination of both factors. Moreover, high correlations between mediators may have introduced multicollinearity, potentially inflating standard errors and reducing model stability. It is also possible that the theoretical structure imposed by the model does not fully reflect the actual causal dynamics among variables, which could lead to distorted path estimates and weakened overall fit. To address these limitations, the global model was subsequently divided into three partial models, each specifying fewer observed variables and fewer estimated paths. This approach allows for a more parsimonious representation of the data and may yield improved model fit by reducing complexity and potential sources of misspecification.

#### Religiosity model

**Fig. 3:**
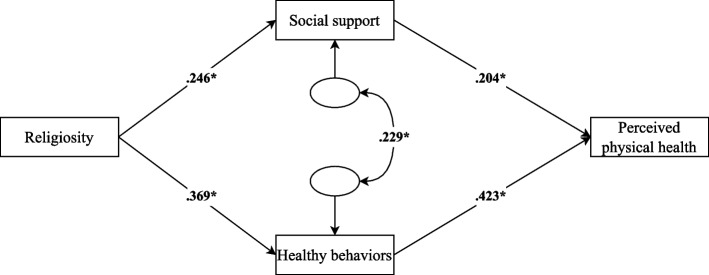
Path model of religiosity predicting perceived physical health via social support and healthy behaviors. Note. The standardized effects between variables are shown. *p < 0.05

The first partial model examined the role of religiosity as the independent variable, with social support and healthy behaviors specified as mediators of its relationship with perceived physical health. This model is presented in Fig. 3.

This model showed significant regression coefficients for all paths, and explained 27.2% of the variance in perceived physical health. However, the model did not demonstrate an adequate fit, suggesting poor model specification: χ^2^ = 364.188, df = 20 (*p* < 0.001); χ^2^/df = 32.6; CFI = 0.759; TLI = 0.278; RMSEA = 0.300, 90% CI [0.240, 0.364]; SRMR = 0.102. While the direct relationships between variables are statistically significant, the model's structure does not fully capture the underlying dynamics.

#### R/S struggles model

**Fig. 4:**
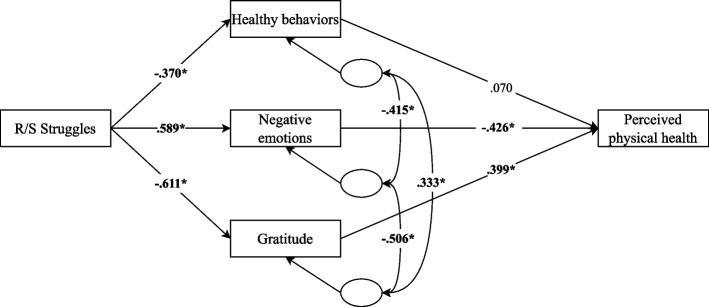
Path model of R/S struggles predicting perceived physical health via healthy behaviors, negative emotions, and gratitude. Note. The standardized effects between variables are shown. *p < 0.05

The second partial model examined the role of R/S struggles as the independent variable, with healthy behaviors, negative emotions, and gratitude as mediators of its relationship with perceived physical health (Fig. 4). All regression paths were significant except for the effect of healthy behaviors on perceived physical health. The model showed an adequate fit (although the chi-square statistic remained significant): χ^2^ = 13.04, df = 2 (*p* < 0.001); χ^2^/df = 6.52; CFI = 0.988; TLI = 0.939; RMSEA = 0.125, 90% CI [0.067, 0.194]; SRMR = 0.022. Indirect effects were also calculated. The standardized indirect effect of R/S struggles through healthy behaviors was non-significant (β = -0.026, *p* = 0.078). In contrast, significant indirect effects were found through negative emotions (β = -0.251, *p* < 0.01) and gratitude (β = -0.244, *p* < 0.01). The total standardized indirect effect was substantial and significant (β = -0.520, *p* < 0.01). These results suggest that higher levels of R/S struggles are associated with poorer physical health, primarily through increased negative emotions and decreased gratitude. This model explained 63.5% of the variance in perceived physical health.

#### Spirituality model

**Fig. 5:**
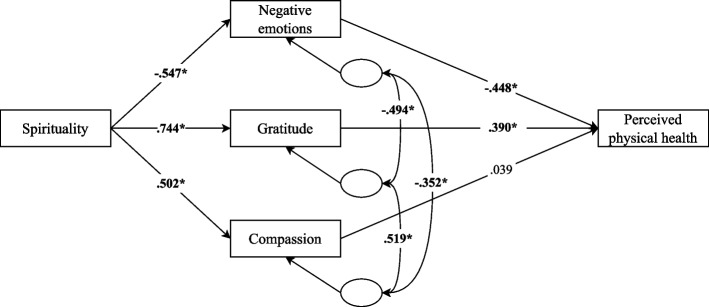
Path model of spirituality predicting perceived physical health via negative emotions, gratitude, and compassion. Note. The standardized effects between variables are shown. *p < 0.05

The third and final partial model examined the role of spirituality as the independent variable, with negative emotions, gratitude, and compassion specified as mediators of its relationship with perceived physical health (Fig. 5). All regression paths were significant except for the effect of compassion on perceived physical health. The model demonstrated an overall acceptable fit, although the chi-square statistic remained significant: χ^2^ = 9.36, df = 2 (*p* < 0.001); χ^2^/df = 4.68; CFI = 0.993; TLI = 0.966; RMSEA = 0.102, 90% CI [0.043, 0.172]; SRMR = 0.017. Indirect effects were also calculated. The standardized indirect effects of spirituality were significant through negative emotions (β = 0.245, *p* < 0.01) and gratitude (β = 0.290, *p* < 0.01), but not through compassion (β = 0.020, *p* = 0.378). The total standardized indirect effect was substantial and significant (β = 0.555, *p* < 0.01). These results suggest that spirituality is associated with better physical health primarily through reduced negative emotions and increased gratitude, while compassion does not appear to play a significant mediating role in this model. The model explained 63.3% of the variance in perceived physical health.

## Discussion

The findings of this study partially support the relevance of the hypothesized variables as pathways of influence in the relationship between religiosity/spirituality (R/S) and perceived physical health in oncology patients. Although the correlational analyses showed significant associations between the studied variables, the main objective was to understand these links in an integrative manner through a joint model. However, the path analysis of this model revealed poor fit indices, despite being supported by theory and previous literature and presenting coherent bivariate associations.

The poor fit of the global model, despite explaining a considerable proportion of variance in perceived physical health, may be attributed to several factors. First, the complexity of the model—combining multiple predictors (religiosity, spirituality, and R/S struggles) and mediators—likely led to an overparameterized structure, which is penalized by fit indices such as CFI, TLI, and RMSEA [79]. Additionally, the simultaneous inclusion of closely related constructs may have introduced multicollinearity, inflating standard errors and reducing the reliability of path estimates. It is also possible that the theoretical structure imposed by the global model failed to accurately capture the distinct mechanisms through which each predictor influences health outcomes, resulting in misspecification. Furthermore, the relatively limited sample size may have been insufficient to support such a complex model, affecting its statistical stability [80].

Another plausible reason for the poor fit of the global model is the weak performance of the religiosity model when examined in isolation. The partial model focused on religiosity showed that, in line with previous studies, religiosity had positive effects on social support [44, 45] and healthy behaviors [30], which in turn were positively related to perceived physical health [28, 40]. Moreover, the model revealed significant indirect effects of religiosity on health through both social support and healthy behaviors. However, despite showing significant regression paths, the religiosity model was the only one of the three partial models that demonstrated poor fit indices. This suggests that the structure proposed for religiosity—its connections with social support and healthy behaviors as mediators—may not have adequately captured the true underlying processes linking religiosity to perceived physical health. One alternative explanation, following Uchino et al. [81], is that social support may act more accurately as a mediator between religiosity and other variables more directly related to physical health, such as healthy behaviors. Furthermore, while the present study used a general measure of social support, the use of specific measures of religious social support [82] might have better captured the influence of religiosity. It is important to consider how perceived physical health was assessed in this study. The physical and functional subscales of the FACT-G [74] were used, which focus on issues commonly associated with illness and treatment, such as pain, fatigue, and nausea. From this perspective, engaging in healthy behaviors may not have a direct impact on the perception of these symptoms, as other variables may play a more immediate role. Moreover, previous research has pointed out that cancer diagnosis and treatment often lead to increased efforts to adopt healthy habits and avoid risky behaviors [83]. In our study, most participants reported high levels of healthy behaviors, which may have produced a ceiling effect, limiting the variability of this variable and making it harder to detect further improvements in perceived physical health related to these habits.

That said, when the suboptimal structure of the partial religiosity model was incorporated into the more complex global model, it may have contributed disproportionately to the overall misfit. In this sense, the religiosity segment may have acted as a source of misspecification, lowering the global model’s performance even if other segments (e.g., spirituality and R/S struggles) were better specified. In contrast, the three partial models—each isolating one predictor and a subset of mediators—offered more parsimonious representations of the data. By reducing complexity and focusing on theoretically coherent pathways, these models achieved better fit and clearer patterns of indirect effects. This suggests that disaggregating the global model was not only methodologically appropriate but also essential for revealing the nuanced roles of religiosity, spirituality, and R/S struggles in shaping physical health.

In this regard, the partial models of spirituality and R/S struggles showed good fit indices and high explained variances for perceived physical health. On the one hand, significant indirect effects of spirituality on physical health were identified through the reduction of negative emotions and the increase in gratitude, while the direct effect through compassion was not significant. On the other hand, higher levels of R/S struggles were associated with an increase in negative emotions and a decrease in gratitude, generating indirect effects on health through both variables. In this latter case, healthy behaviors were not a significant pathway.

The findings related to negative emotions align with Aldwin et al.'s [18] proposal regarding the role of emotional regulation as an underlying mechanism in the relationship between R/S and health, and suggest that the absence of feelings of abandonment and anger toward God, alongside a positive relationship with God, may significantly influence physical health through this emotional variable. In line with Johnson [31], the regulation of negative emotions could be especially important in patients with life-threatening diseases like cancer, as reducing these emotions could have a positive impact on health outcomes.

In fact, in oncological contexts, where patients face elevated levels of stress and suffering, the ability to regulate negative emotions, together with cultivating gratitude, may play a crucial role in the perception of health. In this line, gratitude emerged as a relevant pathway. Specifically, higher spirituality was associated with higher levels of gratitude, which, in turn, was related to a better perception of health. Conversely, higher levels of R/S struggles predicted a decrease in gratitude, which translated into a worse perception of physical health. Following Büssing et al. [84], in populations with physical illnesses such as cancer, feelings of gratitude facilitate perceptions and cognitions that go beyond the illness situation, allowing focus on the positive aspects of the experience. This could influence how patients perceive pain, fatigue, or their ability to lead a normal life, and, therefore, how they perceive their physical health. Previous results by Mills et al. [85] are consistent with this idea, as they observed that gratitude mediated the relationship between spiritual well-being and physical symptoms, such as fatigue or sleep quality.

Unlike negative emotions and gratitude, compassion did not show a significant effect on perceived physical health in the present study, contrary to what has been observed in other research [59]. One possible explanation is that a cancer diagnosis has the potential to generate compassion for others, which plays an important role in post-traumatic growth after diagnosis [86]. In this sense, it is possible that compassion has a clearer impact on relational or emotional dimensions of well-being rather than on self-reported physical health. Therefore, we suggest that future research consider compassion as a moderator of the relationships between R/S and emotions, rather than as a variable that has direct effects on physical health. Moreover, it should be noted that in clinical contexts like cancer, where personal suffering may be particularly high, patients may prioritize self-care strategies over those focused on others, which could limit the direct influence of compassion on their perceived physical state. In this sense, an assessment of self-compassion [87] could be beneficial to detect underlying mechanisms in future research.

In any case, although the results for gratitude and compassion are disparate, this study represents an important attempt to integrate variables typical of positive psychology along with others more studied as pathways between R/S and health. We believe that the Positive Religious and Spiritual Development Theory [46] can be an optimal and compatible framework with other theoretical explanations for understanding the relationships between R/S and health.

In summary, as proposed, religiosity, spirituality, and R/S struggles showed specific effects on health through different psychological pathways. Although these effects were evaluated in independent models—since the joint model did not show a good fit—the comparative analysis allows for identifying common patterns. Specifically, both spirituality and R/S struggles were related to perceived physical health through their opposite impact on two key variables: negative emotions and gratitude. This finding reinforces the utility of Aldwin et al.'s [18] model to decompose the mechanisms involved in the relationship between R/S and health.

This study provides a global and integrative view by analyzing the interactions between different pathways to better understand the relationship between R/S and physical health and well-being in oncology patients. Thus, to provide patients with the best possible care, our results invite health professionals to consider R/S along with physical, psychological, and social aspects in their prevention, care, and intervention plans. For example, practitioners might carefully assess not only the positive but also the negative and conflicting aspects of R/S, as both have been shown to be important for developing a disposition toward gratitude and for reducing negative affectivity, thereby enhancing the well-being of Spanish cancer patients. Similarly, incorporating aspects such as gratitude (to the extent that it is promoted by R/S) in psychological and/or spiritual interventions can lead to very positive outcomes for patients' well-being. In clinical practice, constructs such as gratitude can be therapeutically addressed through interventions like gratitude journaling or making gratitude lists. These interventions aim to improve physical health by reducing pain, physical symptoms, or insomnia [47], and to enhance mental health by decreasing negative emotions, anxiety, and depression [88]. Additionally, given that spiritual care is still insufficiently integrated in many healthcare settings, emphasizing constructs such as gratitude could help bridge that gap by providing concrete, actionable targets for interventions [89]. Overall, acknowledging and working with these positive psychological traits within the context of religiosity and spirituality in clinical care could contribute to a more holistic and effective approach to cancer patient well-being.

### Limitations and future directions

An important limitation of this study is that the proposed joint theoretical model did not present an adequate fit to the data, which has already been discussed previously in relation to structural complexity, sample size, and the specification of the religiosity segment. Given this situation, partial models were analyzed, which offered a better fit and allowed for the identification of differentiated mechanisms for each dimension of R/S. First, further studies with greater statistical power will be needed to address the full model more robustly. In addition, given the apparent relevance of psychological pathways in the oncological context, as well as the exclusion of variables such as social support and healthy behaviors, which penalized the model in this sample, future studies could benefit from the inclusion of relevant variables in disease contexts, such as treatment adherence [27], or other positive psychological variables such as forgiveness, altruism, or hope, which have also been widely linked to R/S [90].

Likewise, a population as specific as Spanish cancer patients limits the cross-cultural generalization of our results and limits its scope to western tradition countries. However, this specificity also proves to be an important approach to the study of psychosocial and behavioral determinants derived from R/S and their influence on the health of cancer patients in a context in which there has been hardly any research.

Additionally, due to the religious tradition that surrounds the context of the study, forms of spirituality that imply the search for and relationship with a divinity -particularly expressed as a relationship with “God”- were considered. We acknowledge that this represents a relatively narrow operationalization of spirituality, which may not fully capture other forms of spiritual experience or connection to the transcendent that do not involve a theistic framework. Future research should address this limitation by including measures that reflect broader and more inclusive understandings of spirituality, in line with the increasing diversity of belief systems observed in the Spanish context [91].

Finally, for feasibility reasons, this study provides cross-sectional data. Both qualitative and longitudinal studies could improve our understanding of the dynamic relationships between R/S and relevant aspects of health and well-being of cancer patients in Spain and elsewhere. Moreover, due to the study design, participants were asked to complete the entire questionnaire in a single session. On average, completing the questionnaire took approximately 15 to 20 min. This approach was necessary for practical reasons, but the considerable number of scales used to assess the variables in the study may have contributed to participant fatigue or a decrease in motivation, which might have affected the quality of the responses. Future research should consider distributing the completion of scales across multiple sessions to mitigate these effects.

## Conclusion

Our study has shed some light on the psychosocial and behavioral pathways underlying the relationships between religion, spirituality, and health in Spanish cancer patients. The empirical data show that the decrease in negative emotions and the promotion of gratitude—fostered by higher levels of spirituality and lower levels of R/S struggles—lead to better perceived physical health in these patients. This suggests that addressing spirituality and R/S conflicts as part of patient care could improve quality of life, particularly in contexts where illness challenges religious and/or spiritual beliefs. However, given that the global theoretical model did not show a good fit, the analysis was carried out through three separate partial models, which, although more parsimonious and better adjusted, limit the integrative interpretation of all dimensions of R/S in a unified framework.

Despite Spain’s strong religious traditions, spirituality and religiosity remain underexplored in healthcare settings—likely due to uncertainty, lack of training, or a misunderstood notion of professional neutrality. The limited integration of these aspects into oncological care suggests a missed opportunity to support patients holistically. Given that R/S can serve both as a source of distress and as a coping resource, it is crucial to equip healthcare providers with the knowledge and tools needed to address these dimensions appropriately. Developing training programs and clinical guidelines could help bridge this gap, ensuring that spiritual concerns are neither dismissed nor imposed but rather respectfully integrated as part of patient-centered care.

A better understanding of the empirical relationships between religion, spirituality, and health can contribute to improving the quality of life of cancer patients, promoting truly holistic care. Identifying these correlations and pathways enables healthcare practitioners to incorporate spiritual care into the evidence-based processes aimed at improving patients’ well-being. This requires not only greater awareness, but also structured training for professionals, interdisciplinary collaboration, and the development of validated interventions that integrate spirituality as a resource. Future research should explore how these approaches can be effectively implemented in different healthcare contexts to ensure that spiritual care becomes an essential component of comprehensive, person-centered treatment.

## Data Availability

The datasets generated and/or analysed during the current study are not publicly available due to patient association data protection policies but are available from the corresponding author on reasonable request.
